# Exploration of cerebral vasospasm from the perspective of microparticles

**DOI:** 10.3389/fnins.2022.1013437

**Published:** 2022-10-28

**Authors:** Yalong Gao, Kai Li, Xiaotian Li, Qifeng Li, Jiwei Wang, Shu Zhang, Jianning Zhang

**Affiliations:** ^1^Key Laboratory of Post-Neurotrauma Neurorepair and Regeneration in Central Nervous System, Ministry of Education and Tianjin Neurological Institute, Tianjin Medical University General Hospital, Tianjin, China; ^2^Tianjin Key Laboratory of Cerebral Vascular and Neurodegenerative Diseases, Department of Neurosurgery, Tianjin Huanhu Hospital, Tianjin, China

**Keywords:** microparticle, cerebral vasospasm, endothelial microparticle, subarachnoid hemorrhage, traumatic brain injury

## Abstract

Cerebral vasospasm is a frequently encountered clinical problem, especially in patients with traumatic brain injury and subarachnoid hemorrhage. Continued cerebral vasospasm can cause cerebral ischemia, even infarction and delayed ischemic neurologic deficits. It significantly affects the course of the disease and the outcome of the patient. However, the underlying mechanism of cerebral vasospasm is still unclear. Recently, increasing studies focus on the pathogenic mechanism of microparticles. It has been found that microparticles have a non-negligible role in promoting vasospasm. This research aims to summarize the dynamics of microparticles *in vivo* and identify a causal role of microparticles in the occurrence and development of cerebral vasospasm. We found that these various microparticles showed dynamic characteristics in body fluids and directly or indirectly affect the cerebral vasospasm or prompt it. Due to the different materials carried by microparticles from different cells, there are also differences in the mechanisms that lead to abnormal vasomotor. We suggest that microparticle scavengers might be a promising therapeutic target against microparticles associated complications.

## Introduction

Cerebral vasospasm (CVS) is a common secondary injury in patients with subarachnoid hemorrhage (SAH) and traumatic brain injury (TBI). Vasospasm occurs in 67% of aneurysmal SAH patients and is symptomatic in 30–40% (Baggott and Aagaard-Kienitz, 2014). Among children with TBI, CVS takes up a high proportion (45.5%) of events affecting the middle cerebral artery (O’Brien et al., 2010). CVS may contribute to delayed ischemic neurologic deficits (DIND) and cerebral infarcts that seriously affect patient outcomes. Nevertheless, the mechanism behind CVS is still confusing.

Cerebral vasospasm is the narrowing of the arteries caused by a persistent contraction of the intracranial blood vessels and consequent changes in hemodynamics, which is mainly due to multiple pathological factors. The diagnosis of CVS is mainly based on the clinical symptoms, signs, and radiographic results (mainly angiography). But there may be a mismatch between radiographic findings and clinical outcomes. Asymptomatic vasospasm means that patients showed no neurological deficits, but vasoconstriction was found on radiography (Aldakkan et al., 2017). Similarly, when neurological deficits and radiographic findings occur simultaneously, it is defined as symptomatic vasospasm (also called DIND) (Francoeur and Mayer, 2016). Among these radiological examinations, digital subtraction angiography is considered the gold standard for diagnosing CVS. Nevertheless, cerebral infarctions caused by tiny vasospasms cannot be detected on radiological examinations. Regarding the mechanism of CVS, previous studies mainly focused on inflammation, smooth muscle contraction, thrombosis, hemostasis and spreading depolarization (Perrein et al., 2015; Geraghty and Testai, 2017). The treatment methods based on these theories are not satisfactory (Daou et al., 2019; Neifert et al., 2021). Therapeutic modalities that simply antagonize vasospasm, such as endothelin blockers and calcium channel blockers, did not improve neurological outcomes. These “unexpected results” suggest that the problem is not so simple and we need a new paradigm to explore the mechanisms of cerebral vasospasm.

Logsdon et al. (2015) concluded that a vasoconstrictor released by damaged pericytes, intracranial pressure spike, and intracranial hemorrhage caused by trauma can induce CVS. They emphasized the important role of vasculature and the neurovascular unit in CVS following TBI. Quintin et al. (2022) proposed that there might be link between the glial-lymphatic system disruption and CVS based on existing studies. Although there is no direct evidence, it provides the direction of subsequent research. There are some reported cases of CVS occurring after surgical resection of intracranial tumor (Bougaci and Paquis, 2017; Agarwal and Dutta Satyarthee, 2019). This is a rare but challenging complication with very poor outcomes and multiple pathogenetic mechanisms contribute to this (Alotaibi and Lanzino, 2013). Reversible cerebral vasoconstriction syndrome (RCVS) is a complex neurovascular disorder and the cerebral vascular tone dysfunction and blood-brain barrier impairment are thought to be involved in RCVS. Previous studies enrich the theoretical basis of CVS, but still cannot fully explain CVS.

Microparticles (MPs, also called microvesicles) with multiple biological effects may link these pathological changes in tandem and there is the latest evidence supporting the direct involvement of MPs in CVS (Wang et al., 2022). Their results demonstrate that brain-derived microparticles (BDMPs) constrict blood vessels depending on their structure. Thus, MPs may serve as both markers and mediators of CVS.

**FIGURE 1:**
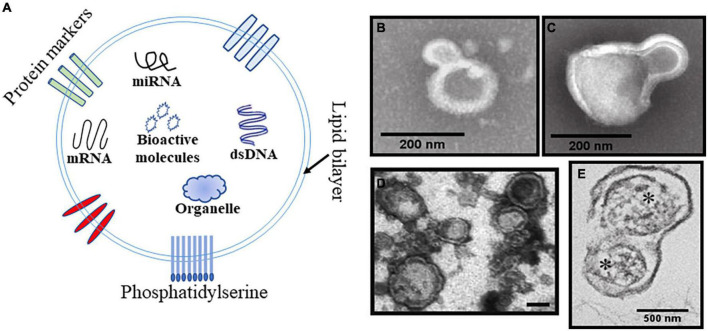
Schematic and representative Transmission electron microscopy (TEM) images of the MPs. **(A)** Schematic diagram of MPs structure including phospholipid bilayer, internal genetic material, bioactive molecules organelles, and exposed phosphatidylserine. **(B,C)** TEM of representative platelet-derived MPs (PMPs) and monocyte-derived MPs produced by the stimulated parent cell. Scale bar is 200 nm. **(D)** TEM image of brain derived MPs (BDMPs) from freeze-thawing injury. Scale bar is 200 nm. **(E)** TEM image of mitochondria (*)-embedded BDMPs detected in plasma samples from TBI mice. Scale bar is 500 nm.

Cellular MPs were first reported by Wolf (1967) as dust produced by platelets. Afterward, the cognition of MPs experienced an iterative process. The essence of MPs is their cell-derived membranous structures that range from 100 to 1,000 nm in diameter (van Niel et al., 2018). Flow cytometry is currently the most widely used technology for MPs research due to its advantages in determining typing, and a few are electron microscopy, mass spectrometry and nanoparticle tracking analysis (NTA). MPs are composed of a phospholipid bilayer and internal genetic material, bioactive molecules and organelles and are considered as an additional mechanism for intercellular communication, allowing cells to exchange these materials (Figure 1A). MPs can also be prepared *in vitro* and used in research (Aleman et al., 2011; Figures 1B,C). Some studies have indicated that circulating MPs contribute to coagulation, apoptosis, oxidative stress, inflammation, and immune regulation (Voukalis et al., 2019). Almost all cell types of the central nervous system (CNS) have been shown to release MPs, which could be important for certain pathophysiological processes (Schindler et al., 2014; Andjus et al., 2020). MPs could cause increased vascular tone, which is observed in many diseases (Tual-Chalot et al., 2012; Han C. et al., 2020). It is therefore meaningful to explain how MPs affect CVS, which could help further research into this condition. In this review, we explore CVS from the perspective of MPs and put forward promising treatment strategies. Research relevant to our topic includes epidemiology of CVS, characterization and dynamics of various MPs following SAH and TBI and their possible mechanisms. Although both belong to extracellular vesicles, exosomes are different from MPs in diameter and biogenesis mechanisms (Xu et al., 2016; Hessvik and Llorente, 2018). Since few studies are focusing on exosome-related vasospasm, it will not be discussed too much in this review.

### Changes in microparticles levels associated with traumatic brain injury and subarachnoid hemorrhage

The increase of MPs in plasma has been reported very early after TBI (Jacoby et al., 2001). MPs from different cell sources can be identified by their specific markers (Table 1). A study including 16 patients with severe TBI found that endothelial-, platelet-, and leukocyte-derived MPs (EMPs, PMPs, and LMPs) were elevated 72 h after injury by flow cytometry. The peak level of endothelial microparticles (EMPs) was ∼7 times higher than the control (Nekludov et al., 2014). However, the relationship between elevated MPs and pathological changes in TBI is not clear. Tian first reported that MPs expressing neuronal or glial cell markers (brain-derived microparticles, BDMPs) were released from injured brain tissue and reached the peak concentration in plasma 3 h after TBI and induced a hypercoagulable state (Tian et al., 2015). Moreover, they prepared BDMPs (Figure 1D) by freezing and thawing the brain and simulated its procoagulant effect. Another study observed the presence of glial fibrillary acidic protein (GFAP) and its breakdown product (GFAP BDP) and UCH-L1 (neuronal cell body biomarker ubiquitin C-terminal hydrolase-L1) in higher concentrations in microvesicles and exosomes from the cerebrospinal fluid (CSF) of patients with TBI compared to control CSF (Karnati et al., 2019). This means that MPs released by the damaged brain tissue enter the CSF. The role of these MPs in CSF seems confused. But as the research progresses, we found that MPs bear numbers of bioactive effects and they can be disseminated, exchanged, and transferred *via* MPs–cell interactions. The activation effect of BDMPs on microglia/macrophages has been observed *in vitro* and may relate to TBI-induced neuroinflammation (Rong et al., 2018). Interestingly, a subset of BDMPs released during the acute phase of TBI contains mitochondria (mtMPs, Figure 1E), which synergize with platelets to facilitate vascular leakage by disrupting the endothelial barrier (Zhao et al., 2016). In addition to being in plasma, MPs can also appear in CSF following TBI, which seems to be associated with poor prognosis and might contribute to poor clinical outcomes (Morel et al., 2008). But the study needs more data to support this conclusion. The mechanism of BDMPs in secondary brain injury after TBI is receiving more attention.

**TABLE 1:** Microparticles marker associated with CVS.

Study type	Year of publication	Biomarker of MPs	Putative role of MPs	Source of MPs
Clinical research	Sanborn et al., 2012	CD235a (erythrocyte); CD66b (neutrophil); CD142 (tissue factor); CD146 (endothelial) vWF	Thrombosis; endothelial dysfunction	Peripheral blood
Clinical research	Lackner et al., 2010	CD31 (endothelial); CD41 (platelet)	Cerebral vasospasm	Peripheral blood
Clinical research	Przybycien-Szymanska et al., 2016	Haptoglobin; fibrinogen α and γ chain, synaptic nuclear envelope protein 2, et al.	Cerebral vasospasm; immune and metabolic processes; cerebrovascular disease states	CSF
Basic research	Bohman et al., 2016	Not given	Cerebral vasospasm	Peripheral blood

Elevated MPs have also been found in SAH patients. Many subtypes of MPs have been reported in the plasma of SAH patients by flow cytometry, including CD105 + and CD62e + (endothelial cell), CD41 + (platelet), CD45 + (leukocyte), CD235a + (erythrocyte), CD66b + (neutrophil), CD145 + (tissue factor, TF), and CD146 + (endothelial cell) (Lackner et al., 2010; Sanborn et al., 2012). A clinical study found that tissue factor-associated MPs and endothelial-associated MPs identified by flow cytometry are elevated in peripheral blood following SAH, and could predict radiographic infarction 14 days after SAH (Sanborn et al., 2012). In their study, MPs showed a potential link to thrombosis, inflammation, and vasospasm after SAH. But, the causal link between MPs and infarction remains uncertain due to the confounding factors in the study. Although studies have confirmed that MPs in CSF elevated, there is still a lack of information on the types of MPs and their temporal dynamics (Przybycien-Szymanska et al., 2016). To further illustrate their biological activities, CSF MPs should be classified not only by their cells of origin but also by their production, structures, and functions.

### Microparticles linked to subarachnoid hemorrhage-related cerebral vasospasm

Microparticles provide transport for multiple types of “molecular cargo” from nucleic acids to lipids and proteins. Przybycien-Szymanska et al. (2016) isolated MPs from CSF for mass spectroscopy studies and revealed marked differential protein expression among CVS patients. Some proteins such as hemoglobin subunit were upregulated in patients who developed post-SAH CVS, which are vasoconstrictor factors. This was the first evidence of differential protein expression in MPs derived from the CSF of CVS patients. Future studies can exploit the advantages of proteomics techniques in finding markers of MP. Saugstad et al. (2017) found some unique RNA contents of MPs in SAH patients compared with other CNS diseases, and these respective RNAs may provide insight into which MPs are uniquely associated with SAH vasospasm. MicroRNAs (miRNAs) can affect the metabolism of cerebral vascular endothelial cells through various mechanisms and finally cause CVS (Gareev et al., 2020).

### Microparticles linked to traumatic brain injury-related cerebral vasospasm

The latest research found that BDMPs injection decreased cerebral blood flow, which is proven to cross the vascular endothelium and contract smooth muscle cells strongly. Furthermore, they could constrict isolated arteries and increase smooth muscle cytoplasmic calcium, which is partially blocked by nimodipine (Wang et al., 2022). This study is the most direct evidence that MPs constrict smooth muscle cells and is very convincing. However, whether this vasoconstriction is endothelium-dependent has not been explored, after all, the endothelium plays a pivotal role in the vascular activity. Endothelin-1 is a key player mediating a strong vasocontractile effect and was increased in the CSF of TBI patients (Michinaga et al., 2020). A previous study demonstrated that elevated serum MPs in a piglet model of TBI led to impaired hypotensive cerebrovasodilation *via* overexpression of tissue plasminogen activator, endothelin-1, and extracellular signal-regulated kinase–mitogen-activated protein kinase (ERK-MAPK) (Bohman et al., 2016). This is another direct evidence that MPs promote CVS in conjunction with endothelin-1. It suggests that the effect of MPs in promoting cerebral vasoconstriction may be indirect by promoting endothelial cells to produce vasoconstrictive substances. However, clinical studies found the protective effect of endothelin antagonists is limited, which suggests that the role of endothelin accounts for a small part of the pathological mechanism. Therefore, it is not difficult to understand that the traditional antispasmodic therapy has little effect because the MPs act on the upstream pathway of vasospasm.

### Specific cell-derived microparticles

#### Endothelial microparticles

The relationship between MPs, endothelial activity and modified vascular tone was elucidated in other studies (Boulanger et al., 2001; Tual-Chalot et al., 2010). Vasomotor function maintenance requires endothelium integrity. Damage to the endothelium may cause abnormal blood vessel contraction. Especially for EMPs, highly increased concentrations were interpreted as indicative of cerebral vascular damage (Mondello et al., 2018). The characteristics of released EMPs appear to change with the functional status of the pathological endothelium. EMPs were shown to be increased in congenital heart diseases and could contribute to endothelial dysfunction *via* P38 MAPK-dependent pathways (Lin et al., 2017). Thus, these EMPs may represent novel biomarkers of endothelial injury and dysfunction. One study suggested that apoptosis in the endothelium of major cerebral arteries may be involved in CVS after SAH, and caspase inhibitors reduced angiographic vasospasm (Zhou et al., 2004). Lackner et al. (2010) studied plasma samples from 20 SAH patients and found that EMPs levels were significantly higher than in healthy controls, and the changing trend of EMPs was consistent with the occurrence of CVS, suggesting it could be used as a predictive factor. Another study also found that EMPs can reflect ischemic events (Jung et al., 2009). However, the underlying mechanism remains unclear among these researches. Increasing evidence suggests that MPs are not simply a consequence of disease; rather, they might be the initiating factor of a variety of pathological and physiological changes (Chironi et al., 2009; Leroyer et al., 2010; Zhao et al., 2017).

#### Red cell-derived microparticles

Erythrocytes regulate vasomotion through modulating oxygen delivery and the scavenging and generation of nitric oxide (NO), which depend on the intracellular hemoglobin (Hb) (Helms et al., 2018). The structure of red cell-derived microparticles (RMPs) is very similar to that of erythrocytes, so it can be inferred that RMPs also have this effect. Indeed, RMPs are potent NO scavengers. The amount of Hb retained in RMPs is ∼20% of the circulating RBCs, and they can scavenge NO (Westerman and Porter, 2016). And Donadee et al. (2011) confirmed similar phenomena. The RMPs reaction with NO is ∼1,000-fold faster than with RBC-encapsulated Hb and is only 2.5–3-fold slower than with cell-free Hb (Said et al., 2017). Plenty of red blood cells (RBCs) entering the CSF is the main pathological manifestation of SAH (Chen et al., 2020). Cell-free Hb released by erythrocytes into the CSF is suggested to cause CVS after SAH (Hugelshofer et al., 2019). However, a question that cannot be ignored is whether lots of RMPs are produced in the subarachnoid space after SAH. Przybycien-Szymanska enriched MPs in CSF from SAH patients with CVS and found that three hemoglobin subunits were upregulated in MPs *via* mass spectroscopy (Przybycien-Szymanska et al., 2016). Many RMPs that may exist in the CSF of SAH patients could be closely related to the occurrence of vasoconstriction, but additional confirmation is needed. RMPs are elevated in circulating blood, while their contribution to CVS is still not clear. Furthermore, there may be multiple mechanisms by which RMPs promote vasospasm. A recent study reported that circulating RMPs could exert significant tension on blood vessels by increasing endothelial oxidative stress in a myeloproliferative neoplasm model (Poisson et al., 2020). However, it is still ambiguous whether RMPs are involved in cerebrovascular vasospasm through oxidative stress. Unlike TBI, RMPs may affect more the SAH-related CVS induced by blood vessel rupture. Because RMPs are abundantly present in the injury sites and body fluids, they should be taken seriously in terms of secondary CVS.

#### Platelet-derived microparticles

Currently, the most concern about the pathophysiological effects of platelet-derived microparticles (PMPs) is related to inflammation, immunity, cardiovascular diseases, hemostasis, and thrombosis (Melki et al., 2017; Lopez et al., 2018). PMPs account for the largest proportion of all MPs in circulation (60–90%) (Ed Nignpense et al., 2019). Their lifespan is shorter, with a half-life of 30 min in mice and 10 min in rabbits (Zaldivia et al., 2017). However, the lifespan of PMPs in humans is unclear. There is also limited research on whether elevated PMPs in circulation are involved in the occurrence of CVS after TBI and SAH and other brain injuries. But the regulating effect of PMPs on vascular tone has been gradually discovered. One study reported that platelets could act as a cellular source of thromboxane A2 with the participation of endothelial cells and induce contractions in the rabbit aorta (Pfister, 1979). It is feasible to speculate that PMPs might similarly modulate the vascular tone. Conversely, PMPs were shown to possess endothelial-repairing capability after arterial injury by enhancing the vasoregenerative capacity of early outgrowth cells (Mause et al., 2010). Therefore, PMPs may exert potential endothelial repair and neuroprotective functions in patients with brain injury (Hayon et al., 2012a,b). These beneficial and unfavorable properties of PMPs increase the complexity and make it challenging for us to understand their effects.

#### Leukocyte-derived microparticles

According to parental cell markers, leukocyte-derived microparticles (LMPs) can be classified based on whether they originate from neutrophils, monocytes/macrophages, or lymphocytes. Although current research mainly focuses on T lymphocyte-derived MPs (TMPs) and they are sparse, their effects on vascular function are complicated. There are conflicting conclusions about the effect of TMP on vascular tone. Safiedeen et al. (2017) found that TMPs caused vasodilation disorders by influencing endoplasmic reticulum stress and mitochondrial function. Coincidentally, MPs derived from human lymphoid T-cell line cultured *in vitro* induced vascular hyporeactivity by upregulating pro-inflammatory proteins (Tesse et al., 2008). TMPs decrease NO production and increase oxidative stress in endothelial cells. Meziani et al. (2006) pointed out that MPs originated most probably from leukocytes were responsible for the cyclo-oxygenase-2 vasoconstrictor component that contributes to preeclampsia. TMPs induce endothelial dysfunction in both conductance and resistance arteries by altering the NO and prostacyclin pathways, and these effects are independent of phosphoinositide 3-kinase and ERK1/2 (Angelillo-Scherrer, 2012). These contradictory results may be caused by the heterogeneity of TMPs, but this makes TMPs research more meaningful. Different from the above mechanisms, another study demonstrated that monocyte/macrophage-derived MPs additionally induced brain endothelial cells to undergo vesiculation and produce EMPs (He et al., 2017). This suggests that MPs may have the characteristics of cascade amplification, which makes it difficult to understand how they affect cerebral artery tone. Indeed, this conclusion was also verified in another study (Zhao et al., 2020). However, the role of peripheral inflammatory cells in the CNS is still controversial and we should elucidate that how these LMPs emerged. The question that LMP in cerebrospinal fluid is secreted by infiltrating peripheral inflammatory cells or by newly activated microglia in the brain will help us understand the role of the LMPs in CVS.

#### Brain tissue (neuronal and glial) derived microparticles

Brain-derived microparticles can be distinguished based on surface markers and cargo corresponding to their parent cell. After acute brain injury, neurons, microglia, astrocytes, and oligodendrocytes released MPs involved in pathological processes and can be used as biomarkers (Ollen-Bittle et al., 2022). The current study focused on the effects of other types of BDMPs on neurons including changes in their morphology and synaptic plasticity (Pistono et al., 2020). In addition, glial cell-derived MP may contribute to neuroinflammation (Ruhela et al., 2021). The effect of BDMPs on vascular tone has not received enough attention and direct evidence is sparse. So far, the results of Wang et al. (2022) directly demonstrated the contractile effects of BDMPs on smooth muscle cells and cerebral vessels. This result opens new perspectives for future research.

### Microparticles induced cerebral vasospasm *via* other pathological processes

Several groups reported that multiple pathological changes including neuroinflammation (Zheng and Wong, 2017), coagulopathy (Amin-Hanjani et al., 2004; Han X. et al., 2020), oxidative stress (Karaoglan et al., 2008), and damaged vascular endothelium (Peeyush Kumar et al., 2019) after brain injury could be involved in CVS occurrence and development. Interestingly, MPs can be found in all these pathological processes. A study found that BDMPs activate microglia leading to the release of pro-inflammatory mediators such as IL-1β, TNF-α et al. (Rong et al., 2018). Another study confirmed that activated microglia-derived MPs independently initiate inflammatory responses and contribute to progressive neuroinflammatory response in the injured brain after TBI (Kumar et al., 2017). These increased levels of inflammatory mediators may be involved in cerebral vasospasm (Eisenhut, 2014). This suggests that MPs may mediate CVS through inflammatory pathways indirectly. But direct evidence is needed to confirm this. In addition, MPs have been suggested to contribute to CVS through other mechanisms such as procoagulant (Boettinger and Lackner, 2015). These secondary pathological changes are connected to MPs at one end and CVS at the other end.

### Microparticles-targeted therapy

As Przybycien-Szymanska pointed out, MPs may be the thread that ties together the diverse theories regarding the process of vasospasm (Przybycien-Szymanska and Ashley, 2015), suggesting they could be an initiating factor of secondary injury. Their effects on CVS may be complicated and depend on a direct and/or indirect pathway (Przybycien-Szymanska and Ashley, 2015). Regrettably, there is still a lack of more evidence and unequivocal mechanisms for the effects of MPs on vasospasm following brain injury. Given the complexity of CVS, treatments that only target a specific mechanism may not yield satisfactory results. The presence of negatively charged phosphatidylserine (PS) on the surface is a common feature of all MPs and could serve as a target to block its effects. Zhou demonstrated that lactadherin (milk fat globule epidermal growth factor 8), an MP-scavenging protein, could significantly improve coagulopathy caused by MPs in TBI mice by enhancing PS-mediated phagocytosis (Zhou et al., 2018). This is the first time that the scavenging effect of lactadherin on MPs has been used to treat secondary damage caused by MPs, and it has shown good results. Several molecules have been shown to participate in the clearance of apoptotic cells, such as Del-1 (developmental endothelial locus-1) (Hajishengallis and Chavakis, 2019), Gas-6 (growth arrest-specific gene 6) (Bellido-Martín and de Frutos, 2008), and annexin V. They could also be used to clear MPs. Because MPs share a key structural element with apoptotic cells (mainly surface exposure of anionic PS), they could be similarly recognized by annexin V (Zhao et al., 2016). Importantly, these proteins have been identified to exert significant protective effects in other disease models and showed promise, although they are not used to link MPs clearance (Liu et al., 2014; Hajishengallis and Chavakis, 2019; Mui et al., 2021). Next, the role of these proteins in alleviating MPs-induced CVS needs to be validated. All of them are endogenous plasma proteins, which can fundamentally reduce or even eliminate pathological damage caused by MPs.

## Conclusion

Cerebral vasospasm is a highly complex, poorly understood event that exacerbates neurologic outcomes. Mechanisms behind CVS are known to point to a variety of pathological processes but treatments targeting these pathways have been less effective. These “unexpected results” suggests that we need to explore the mechanism of CVS from a new perspective. The emergence of MPs provides us with a new clue to figure out CVS.

The common characterization of the MPs is their dynamic properties in biological fluids, where they can interact with cells in contact. And most MPs are thought to express phosphatidylserine, which has a strong procoagulant effect. The effects of the MPs from different origins varies greatly. EMPs is more associated with abnormal vascular activity resulting from endothelial injury. The effect of RMPs on vascular activity was reflected in the regulation of vasodilator substances such as NO (Said and Doctor, 2017). The roles of other blood-cell derived MPs’ are complex involving multiple pathways, and they showed both beneficial and deleterious effects.

Although increasing studies have shown that MPs may be involved in CVS, we don’t know whether MPs are the most important initiating factor. And are there any difference in the MPs mechanism in CVS between TBI and SAH? The results of our research are limited and we are not able to show detailed mechanisms due to the early stage of this research. Further studies are needed to confirm these hypotheses and investigate how MPs can cause CVS in conditions like TBI and SAH. MPs contain a variety of biologically active substances such as matrix metalloproteinases and reactive oxygen species, which make their role as functional mediators more complex. It is difficult to block these intricate processes with a single treatment for inflammation, oxidative stress, or other targets. Additionally, MPs could serve as therapeutic targets for secondary injuries. Because of the differentiated structural and multidimensional activities of MPs, there are still outstanding problems to be solved in applying basic research to clinical translation. The foremost problem is to characterize the subtypes of MPs and biologically active substances. By the increasing biomolecules identified on/in MPs, valuable clues will appear. The second is to block interactions between MPs and target cells. Efficient and accurate MPs scavenging protein needs to be paid attention to. Another question of whether exosomes similar to MPs have similar effects also deserves to be answered. Two ways might make sense. First, proteomics technology may provide support to identify pathogenic factors contained by MPs. Second, it may be promising to develop the MPs scavenging proteins to prevent CVS. This approach is worth trying and might work for all excessive hazardous MPs because it depends on the presence of PS rather than biomarkers.

## Author contributions

SZ and JZ: conception and design of the manuscript. YG: drafting of the manuscript. YG, KL, XL, QL, and JW: critical revision of the manuscript for important intellectual content. KL, XL, QL, and JW: manuscript supervision. YG, KL, XL, QL, JW, SZ, and JZ: final approval of the revised manuscript. All authors contributed to the article and approved the submitted version.
